# Supplementary material for: Dynamics of genome reorganization during human cardiogenesis reveal an RBM20-dependent splicing factory
Source: Nat Commun. 2019 Apr 4;10:1538. doi: 10.1038/s41467-019-09483-5 (PMC6449405; doi:10.1038/s41467-019-09483-5)
Supplement: Supplementary file 1 — Supplementary Information [file 41467_2019_9483_MOESM1_ESM.pdf]

Dynamics of genome reorganization during human cardiogenesis reveal an RBM20-dependent splicing factory

Bertero, Fields et al.

Supplementary Information

<u>Guide to content:</u>

Supplementary Note 1

Supplementary Figures and Figure legends

Supplementary Figure 1. Validation of hESCs and cardiac differentiation.

Supplementary Figure 2. Genome-wide contact maps across differentiation.

Supplementary Figure 3. Intra-chromosomal interactions vary over differentiation by compartment.

Supplementary Figure 4. Expression changes are related to A/B compartment changes.

Supplementary Figure 5. hESCs and hiPSCs show similar changes in chromatin organization during cardiac differentiation.

Supplementary Figure 6. TADs are dynamically regulated across differentiation.

Supplementary Figure 7. Local accessibility is dynamic across differentiation and reflects cell type specific transcription factors.

Supplementary Figure 8. Cardiac specific genes are enriched in B to A regions.

Supplementary Figure 9. Proximity between *TTN* and RBM20 target genes depends on *TTN* transcription and RBM20 expression.

Supplementary Figure 10. Generation of gene edited hESC-CM to investigate the mechanisms regulating *TTN trans* interactions.

Supplementary Figure 11. Uncropped gel images.

Supplementary Tables

Supplementary Table 1. Hi-C sequencing statistics.

Supplementary Table 2. HiC-Rep scores between samples.

Supplementary Table 3. RNA-seq sequencing statistics.

Supplementary Table 4. Biological process gene ontology terms associated with differentially expressed genes peaking in cardiomyocyte stage.

Supplementary Table 5. Biological process gene ontology terms associated with differentially expressed genes peaking in cardiomyocytes in regions of the genome that transition B to A.

Supplementary Table 6. Jaccard index scores for TAD boundary overlap**.**

Supplementary Table 7. ATAC-seq sequencing statistics.

Supplementary Table 8. Biological process gene ontology terms associated with differentially expressed genes peaking in cardiomyocyte stage and *trans*-associated with *TTN*.

Supplementary Table 9. Primer sequences.

Supplementary References

Supplementary Note 1

We also assessed chromatin dynamics in a second pluripotent stem cell line of distinct sex and origin compared to female RUES2 hESCs. For this, we performed *in situ* DNase Hi-C at various time points of cardiac differentiation of WTC11 human induced pluripotent stem cells (hiPSCs), a commonly used line derived from reprogramming of skin fibroblasts from a healthy male donor[1]. Differentiating hiPSCs show the expected progression of lineage marker expression and result in highly pure hiPSC-CM (Supplementary Figure 5a-b). Of note, however, expression of a number of cardiac factors is less robust than observed for hESC-CM (Supplementary Figure 5a), indicating that cardiac maturation is slower in this hiPSC line. Accordingly, while compartment changes for MES and CP genes were remarkably similar, a subset of compartment transitions specific to the CM stage were only partially complete in hiPSC-CM (Supplementary Figure 5c-d). This indicates that similarly to gene expression and chromatin epigenetic changes, nuclear architecture dynamics can be influenced by the variability in the differentiation capacity of various hPSCs[2]. Nevertheless, we noted that the global patterns of chromatin organization change observed in hESCs were largely reproduced in hiPSCs (Supplementary Figure 5c-e), indicating that these are conserved across multiple hPSC types of different origin and sex. Considering the stronger cardiogenic potential of RUES2 hESCs, we focused our subsequent analyses on this cell line.

Supplementary Figures and Figure Legends

Supplementary Figure 1

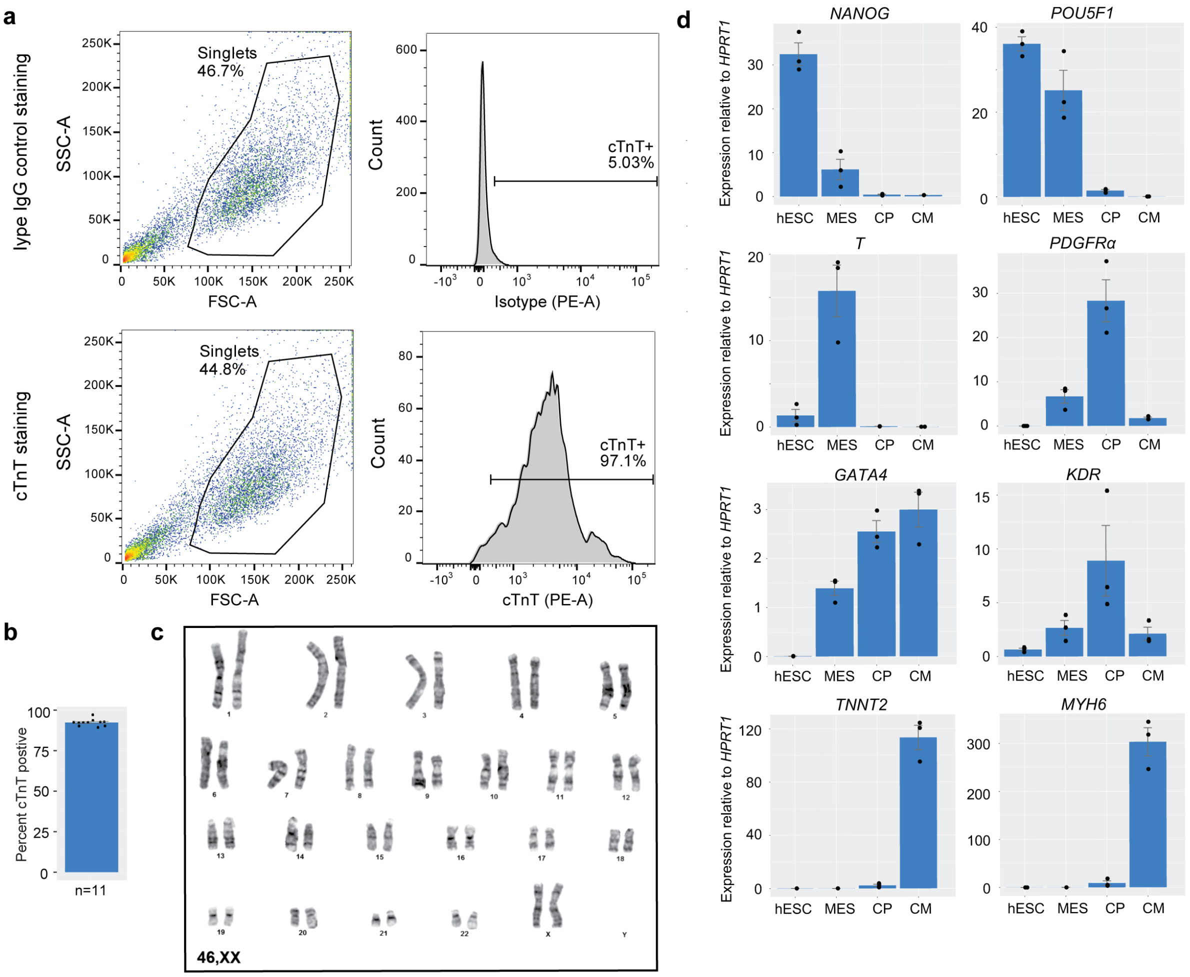

Supplementary Figure 1. Validation of hESCs and cardiac differentiation. (**a**) Representative flow cytometry plot of staining for TNNT2 (cTnT) of day 14 hESC-derived CMs. The gating strategy based on isotype IgG negative control staining is indicated. (**b**) Quantification of percentage cTnT positive in 11 independent differentiations; mean ± s.e.m. is shown. All are greater than 89% cTnT positive. (**c**) Karyotype analysis of RUES2 hESCs showing normal 46 X,X pattern. (**d**) RT-qPCR of stage specific markers of differentiation. mean ± s.e.m. is shown; n = 3 independent differentiations. Source data are provided as a Source Data file.

Supplementary Figure 2

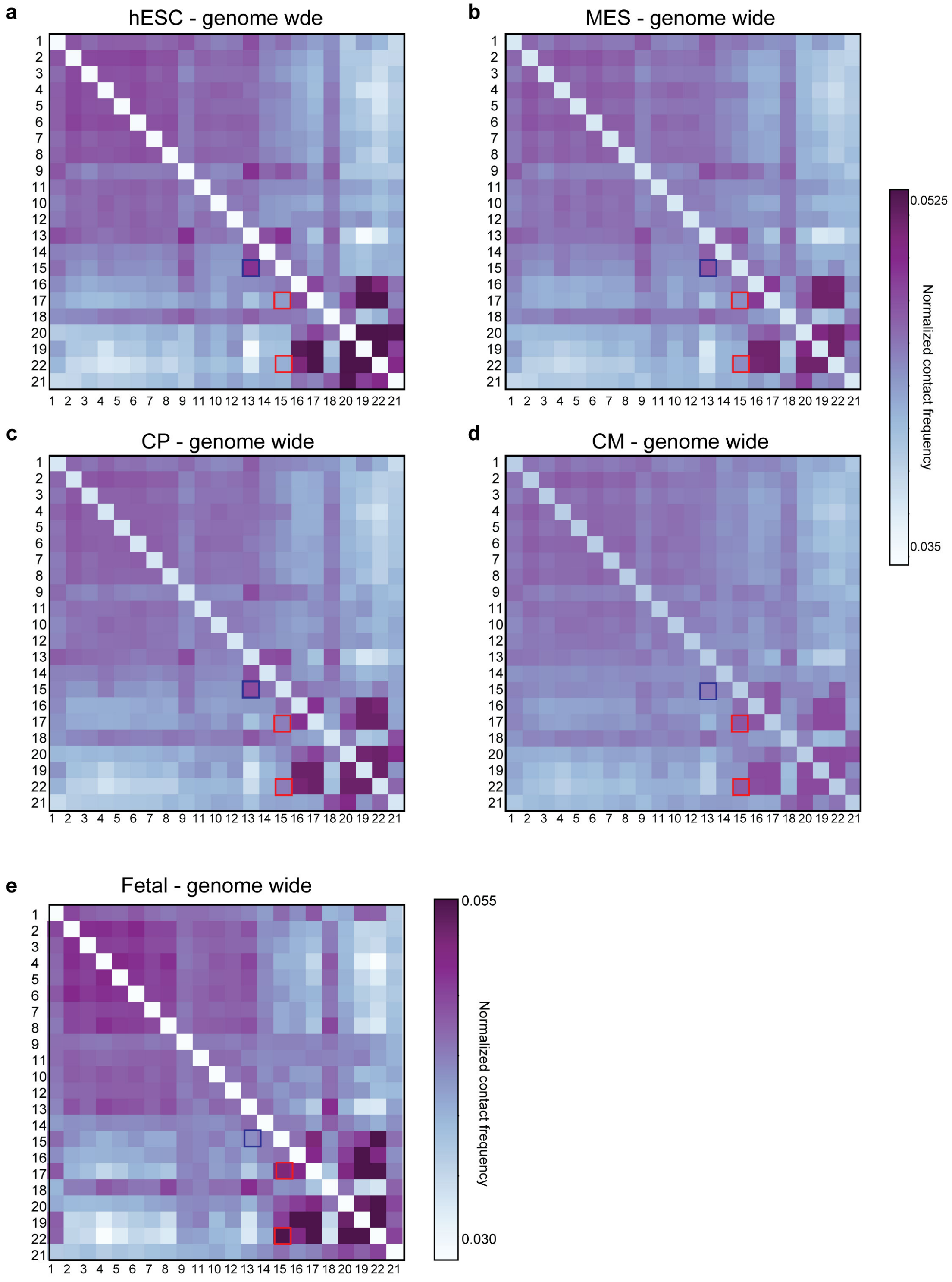

Supplementary Figure 2. Genome-wide contact maps across differentiation. (a-e)

Heatmap of genome-wide contact matrices between chromosomes for each time point across

differentiation and fetal heart. Blue box marks chromosome pair with decreasing signal. Red boxes mark chromosome pairs with increasing signal across differentiation. Few whole chromosome changes are observed across time points save for chromosome 15, which increases its association with the smaller chromosomes in CMs.

Supplementary Figure 3

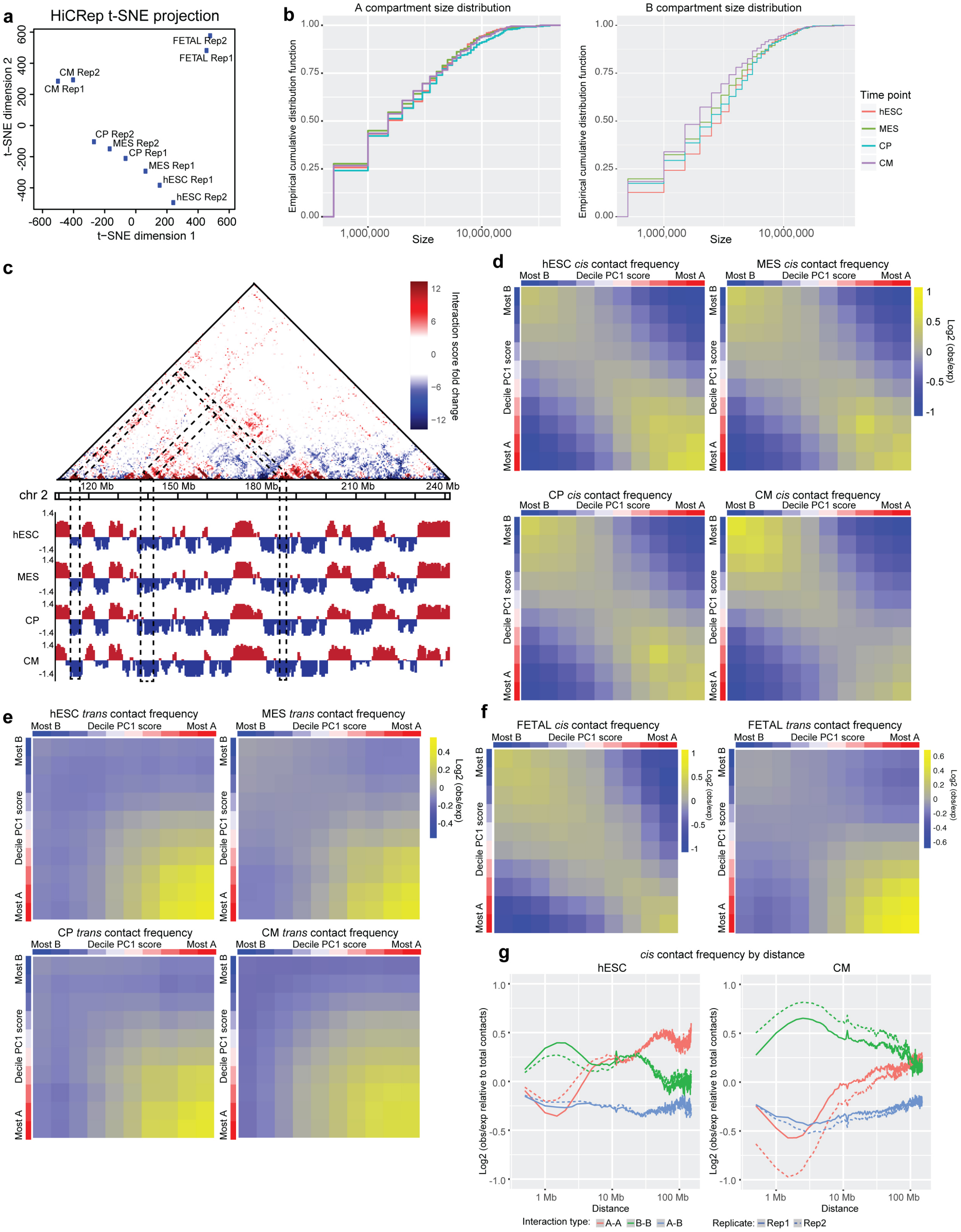

Supplementary Figure 3. Intra-chromosomal interactions vary over differentiation by compartment. (**a**) t-SNE projection of HiC-Rep scores for replicates across differentiation and fetal heart. (**b**) Empirical cumulative distribution function of sizes of compartments across differentiation. Bins were merged to generate sizes of consecutive A or B domains. (**c**) Delta heatmap of the contact matrix of a region of chromosome 2. Dotted lines highlight contacts between B compartment regions. (**d-f**) Compartmentalization saddle plots. Bins were divided in ten deciles and average normalized contact frequency was calculated genome-wide. Normalization was calculated based on all bins at a given distance for *cis* interaction. (d) *cis* contacts across differentiation. (e) *trans* contacts across differentiation. (f) *cis* and *trans* contacts for fetal heart samples. (**g**) Distance plot of A-A, B-B and A-B interactions for hESC and CM, values are normalized to all contacts at a given distance (raw plot from Fig. 1j). Source data are provided as a Source Data file.

Supplementary Figure 4

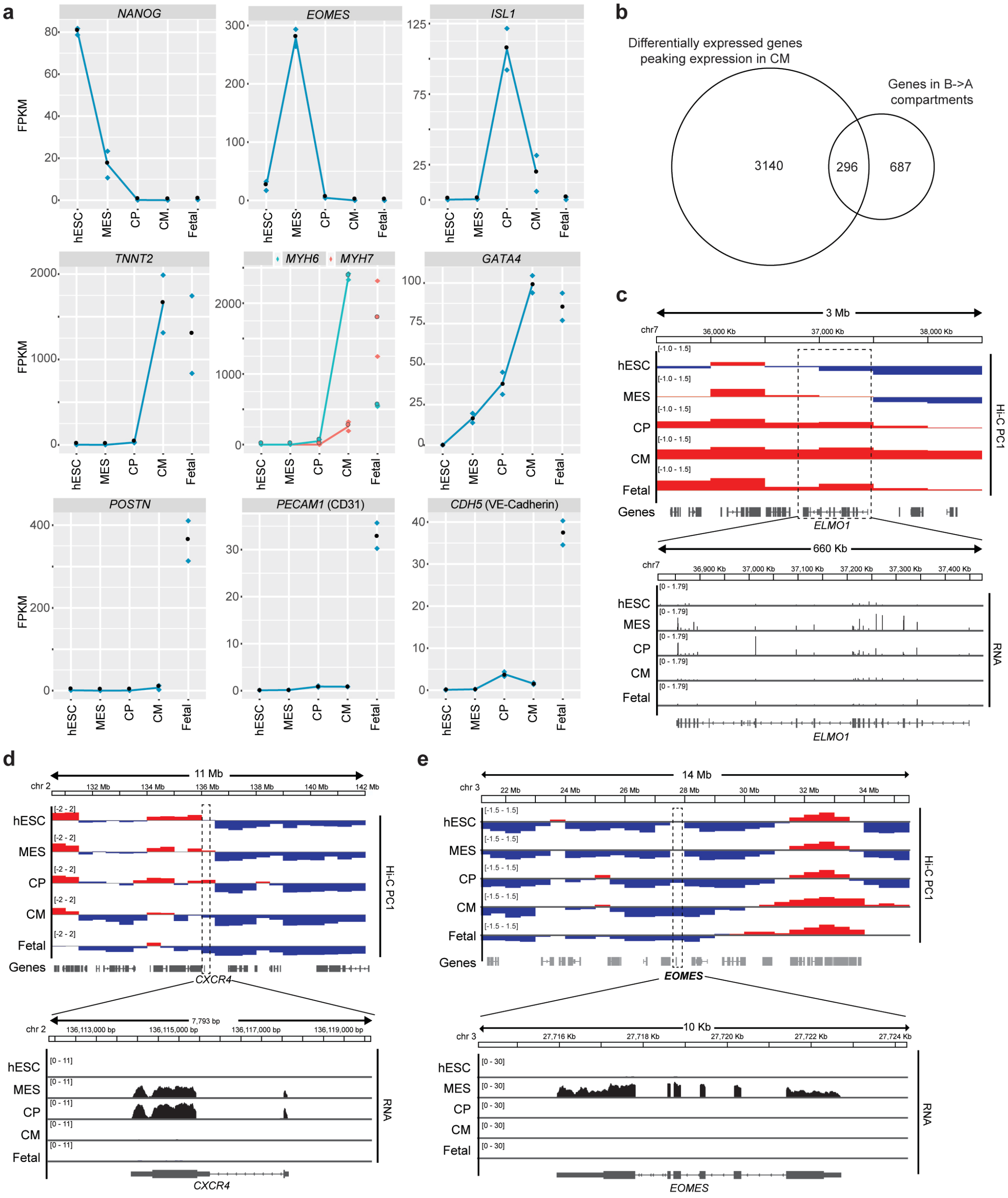

Supplementary Figure 4. Expression changes are related to A/B Compartment changes. (**a**) Expression values for stage-specific and tissue-specific markers. Cardiac specific markers are expressed in CM and fetal heart, while fetal heart expresses markers of both cardiac fibroblasts (*POSTN*) and endothelial cells (*PECAM1* and *CDH5*). (**b**) Overlap between

differentially expressed genes peaking expression in CM and genes found in chromatin domains transitioning from B to A as hESCs differentiate into CM. (**c-e**) Gene tracks of Hi-C PC1 and RNA-seq reads of (c) *ELMO1*, (d) *CXCR4*, (e) *EOMES*. Source data are provided as a Source Data file.

Supplementary Figure 5

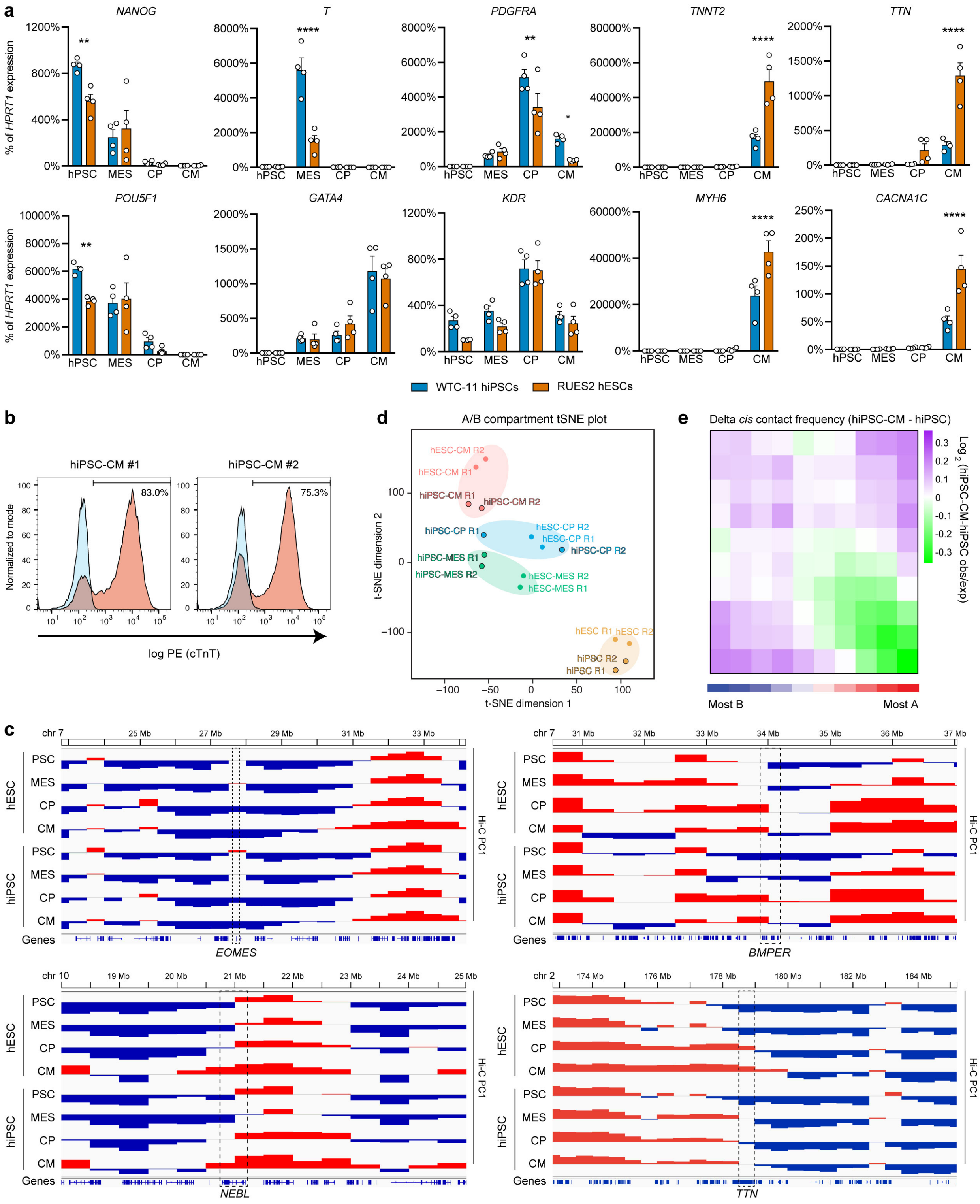

Supplementary Figure 5. hESCs and hiPSCs show similar changes in chromatin organization during cardiac differentiation. (**a**) RT-qPCR at the indicated time points of

hESC or hiPSC differentiation into cardiomyocytes (see Fig. 1a). PSC: pluripotent stem cell (day 0); ME: mesendoderm (day 2); CP: cardiac progenitor (day 5); CM: cardiomyocyte (day 14). Expression is relative to the housekeeping gene *HPRT1* and mean ± s.e.m. is shown. n = 4 independent differentiations. p-values are calculated by two-way ANOVA followed by Holm-Sidak's multiple comparisons for hiPSC vs hESC; * < 0.05; ** < 0.01; **** < 0.0001. (**b**) Flow cytometry for TNNT2 (cTnT) in CM derived from the two independent hiPSC differentiations analyzed by *in situ* DNase Hi-C. (**c**) PC1 scores for 500 Kb-wide genomic bins computed from the Hi-C contact map at the indicated time points of hPSC differentiation; A and B compartments are shown in red and blue, respectively. Representative ME-, CP-, and CM-peaking genes that show similar compartment transitions in hESCs and hiPSCs are reported (*EOMES*, *BMPER* and *NEBL*, respectively). *TTN* is an example of a compartment transition that is not completed in hiPSC-CM by day 14 of differentiation, in line with its lower expression compared to hESC-CM (panel a). (**d**) t-SNE plot of PC1 scores. Samples at the same stage of differentiation cluster closely and are grouped within ovals. R1/2 = first/second biological replicate. (**e**) Delta compartmentalization saddle plot in *cis* contacts hiPSC-CM vs hiPSCs. Bins were assigned to ten deciles based on PC1 score, average observed/expected distance normalized scores for each pair of deciles were calculated. Source data are provided as a Source Data file.

Supplementary Figure 6

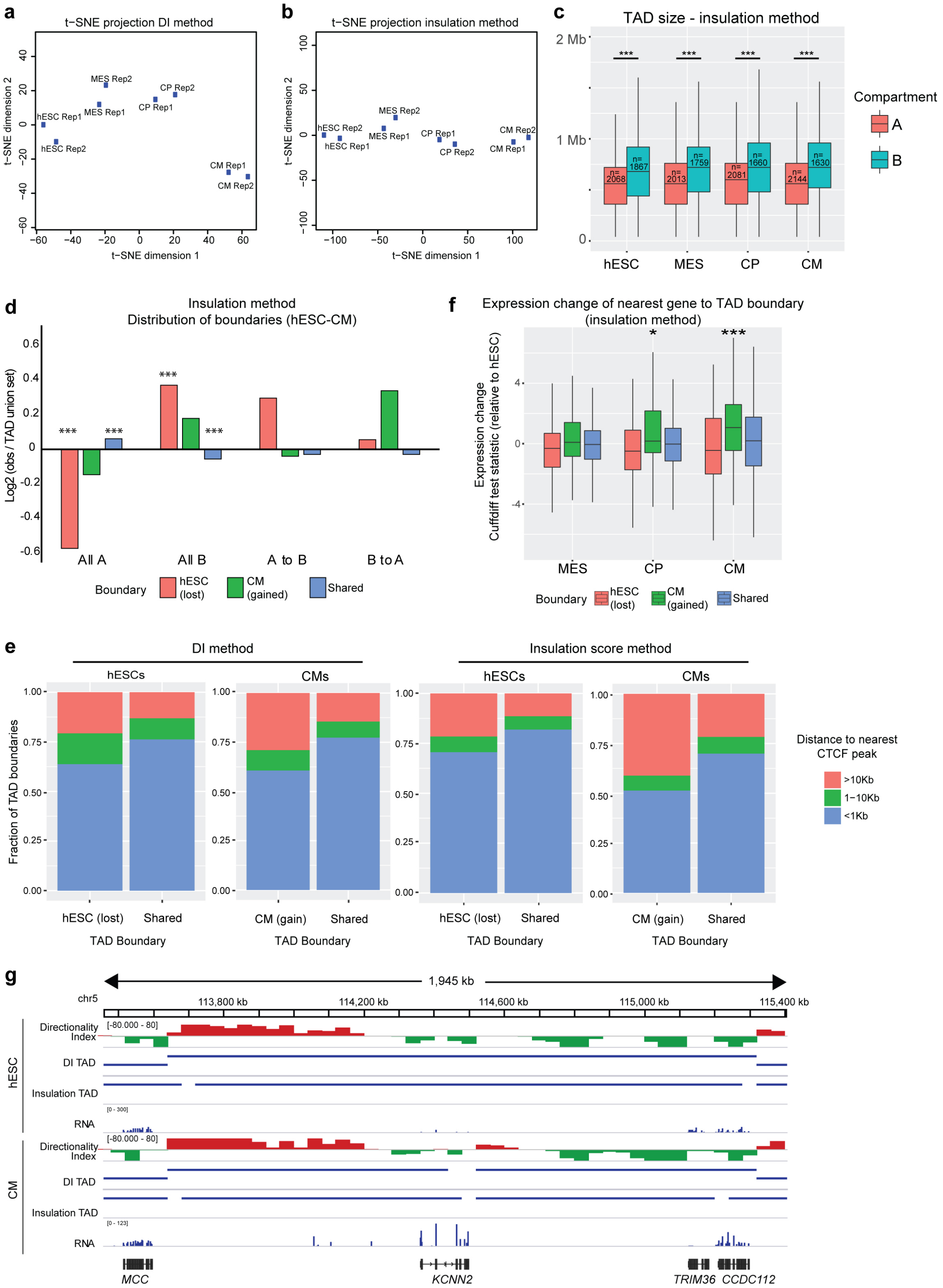

Supplemenary Figure 6. TADs are dynamically regulated across differentiation. (**a-b**) t-SNE projection of Jaccard distance for TAD boundaries based on (a) DI method and (b)

insulation score method across differentiation. Jaccard distance is calculated as 1-(intersection/union) for TAD boundaries within 80 Kb. TAD boundaries cluster by replicate. (**c**) TAD size and number within A and B compartments across differentiation for insulation method. Boxplots present the median and 25th and 75th percentile, with the whiskers extending to 1.5 times the inter-quartile range. n represents the number of TADs. n = TAD number; p-values by Wilcoxon test, *** < 0.001. (**d**) Enrichment of TAD boundaries between hESC and CM state within A/B compartment dynamics for insulation method. Log_2 values are observed/TAD union set; p-values by chi-squared test for the indicated TAD boundary set overlaps; * < 0.05. (**e**) Distance between TAD boundaries and CTCF peaks. (**f**) Expression of nearest genes to TAD boundaries that are either stage specific or shared between hESC and CM for insulation method. Box and whisker plots as in panel c. n =127 genes for hESC (lost) boundaries, 85 genes for CM (gained) boundaries, and 1477 genes for shared boundaries; p-values by one-sample, two-sided t-test relative to a hypothesized mean value of 0 (no expression change vs hESC), * < 0.05, *** < 0.001. (**g**) Gene track of DI score, DI-determined TADs, insulation score-determined TADs, and RNA-seq data for the *KCNN2* locus in hESCs and CM. Source data are provided as a Source Data file.

Supplementary Figure 7

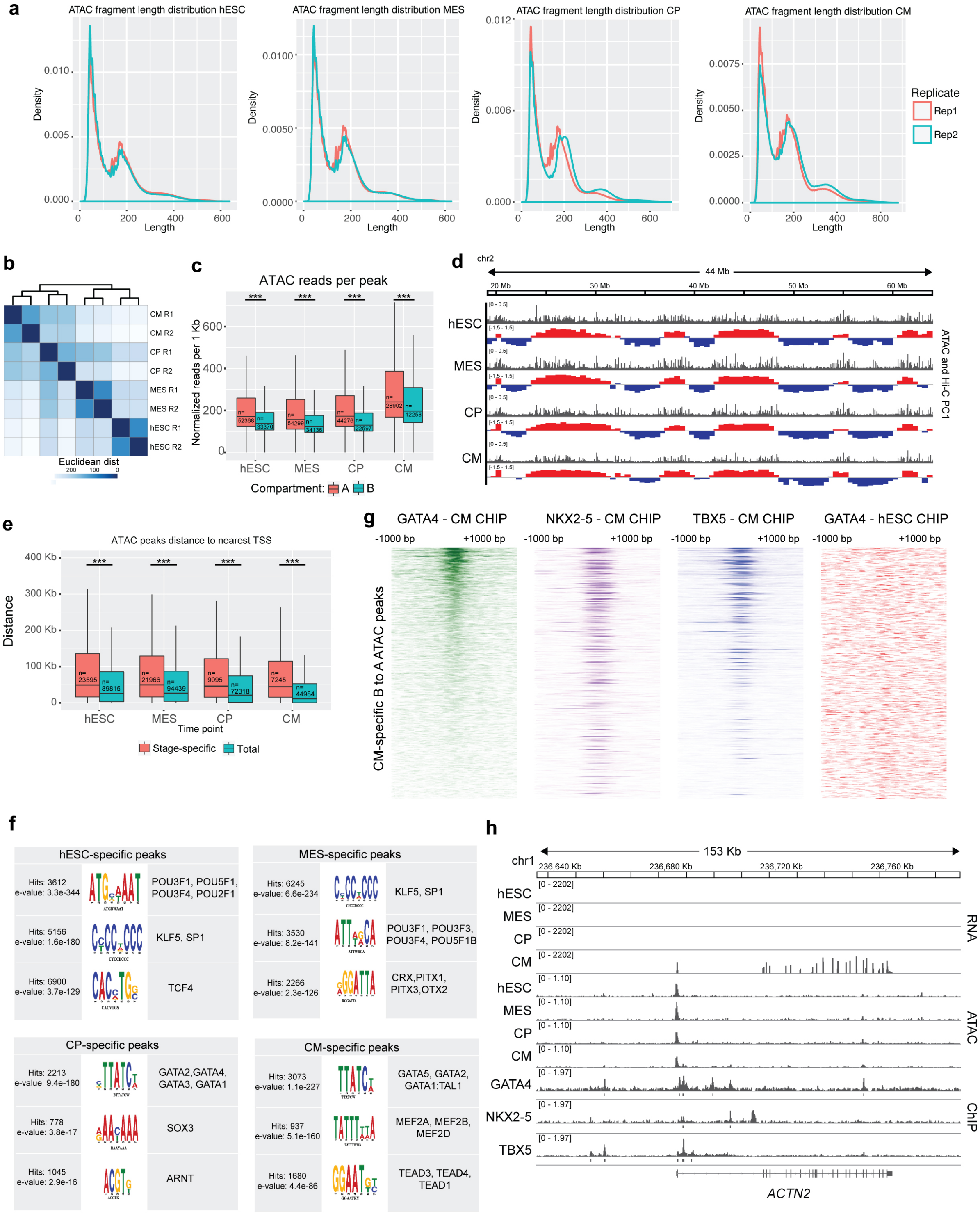

Supplementary Figure 7. Local accessibility is dynamic across differentiation and reflects cell type specific transcription factors. (**a**) Density plot of ATAC read pair sizes

across differentiation for both replicates. (**b**) Heatmap of the correlation of ATAC reads across all ~138,000 peaks in the union set. Clustering pairs replicates and order the samples by differentiation state. (**c**) Normalized reads per 1 Kb for ATAC hotspots within A or B compartment across differentiation. Boxplots present the median and 25th and 75th percentile, with the whiskers extending to 1.5 times the inter-quartile range. n = ATAC peaks number; p-values by Wilcoxon test, *** < 0.001. (**d**) Gene track across a stretch of chromosome for ATAC and A/B compartment, showing enrichment of reads within A compartment compared to B. (**e**) Distance to nearest TSS between stage-specific peaks and total peaks across differentiation. Box and whisker plots as in panel c. n = ATAC peaks number; p-values by Wilcoxon test, *** < 0.001. (**f**) Top enriched motifs identified within stage specific peaks across differentiation, as determined by DREME and TOMTOM, including POU family (OCT4) in hESC and MES, GATA family in CP and CM, and MEF family in CM. (**g**) ChIP-seq reads across CM-specific ATAC in B-A regions for GATA4, NKX2-5, TBX5 (CM) and GATA4 (ESC). (**h**) Gene track of RNA-seq, ATAC-seq, and ChIP-seq across the *ACTN2* locus. Source data are provided as a Source Data file.

Supplementary Figure 8

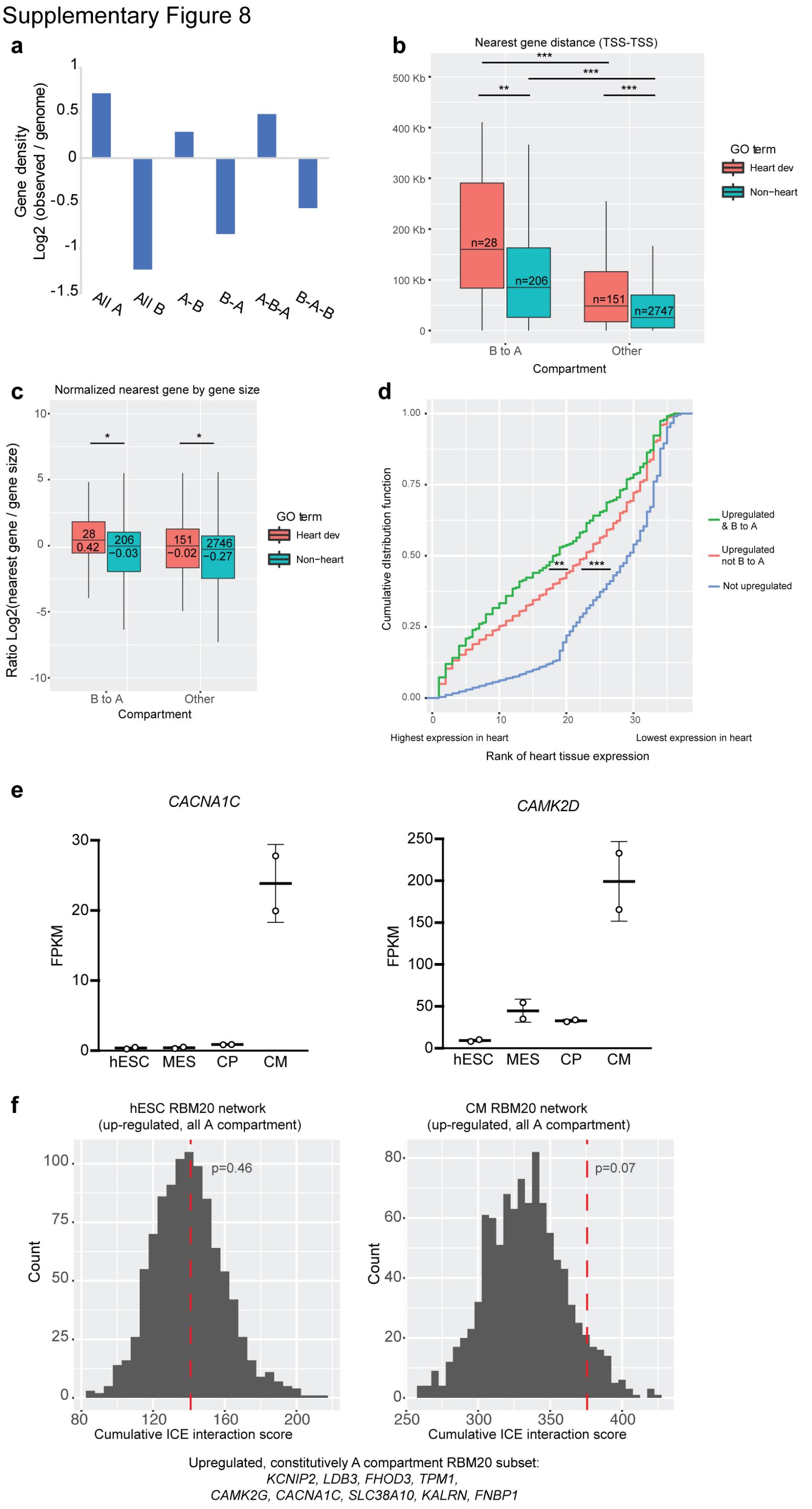

Supplementary Figure 8. Cardiac specific genes are enriched in B to A regions. (**a**) Plot of gene density for compartment dynamics across differentiation. Log_2 observed/expected is

normalized to genome fraction per compartment type. (**b**) Nearest gene distance (TSS-TSS) for upregulated genes peaking in CM stage subdividing by B to A compartment and heart development genes (GO term). Boxplots present the median and 25th and 75th percentile, with the whiskers extending to 1.5 times the inter-quartile range. n = gene number; p-values by Wilcoxon test, ** < 0.01, *** < 0.001. (**c**) Log_2 ratio of distance to the nearest gene divided by gene size for upregulated genes peaking in CM stage subdividing by B to A compartment and heart development genes (GO term). Box and whisker plots as in panel b. Gene number and average log_2 ratio are indicated (top and bottom, respectively); p-values by Wilcoxon test, * < 0.05. (**d**) Cumulative distribution plot of the rank of heart tissue expression for each gene based on RNA-seq data from Protein Atlas. 1 indicates highest relative expression across tissues is in heart. Subdivided by upregulated genes peaking in CM stage in B to A compartment or not and non-upregulated genes. p-values by K-S test, ** < 0.01, *** < 0.001. (**e**) FPKM values for RBM20 target and *TTN trans*-associated genes *CACNA1C* (chr 12) and *CAMK2D* (chr 4) during differentiation. (**f**) Cumulative score of *trans* associations between upregulated and constitutive A compartment RBM20 target genes in hESC and CM, indicated by red line, compared to a background model of 1,000 random permutations of all upregulated, constitutive A compartment genes. The resulting random shuffling p-value is indicated. Source data are provided as a Source Data file.

Supplementary Figure 9

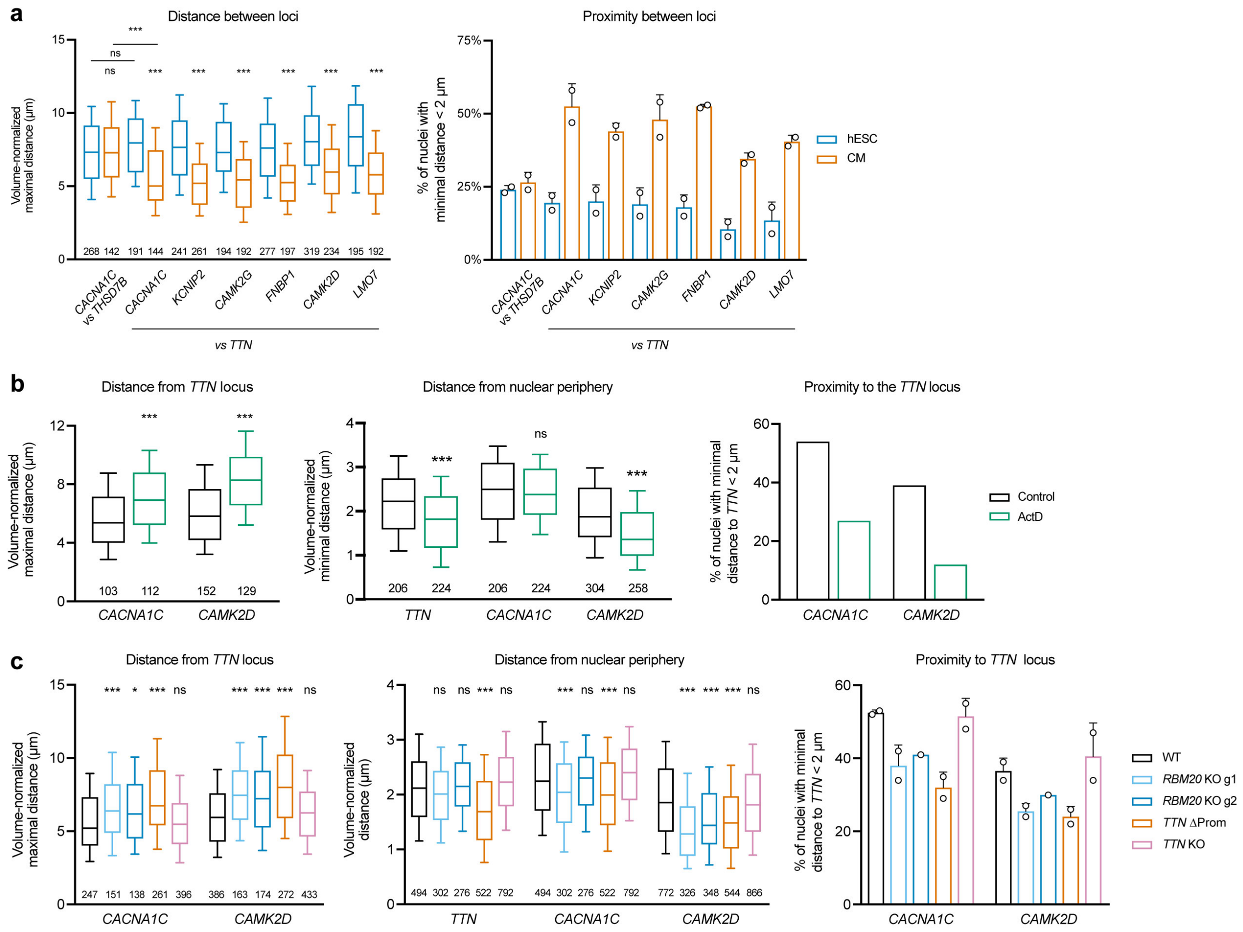

Supplementary Figure 9. Proximity between *TTN* and RBM20 target genes depends on *TTN* transcription and RBM20 expression. (**a**) On the left, normalized maximal distance per diploid cell between the indicated loci in hESC vs hESC-CM (the number of cells is indicated). Box and whiskers plots present aggregated data from two independent cultures, and indicate median, 25th and 75th percentile, and the 10-90 percentile range. p-values by Kruskal-Wallis test followed by Dunn's multiple comparisons vs hESC (unless otherwise indicated); ns ≥ 0.05; *** < 0.001. On the right, proximity between the indicated loci in individual cells (defined as the minimal distance between the centers of the 1 µm-wide FISH spots being less than twice their diameter). Mean ± standard deviation from two independent cultures; no statistical analysis. (**b**) On the left and right, same graphs as in panel a but for hESC-CM maintained in standard

culture conditions (Control) or treated with 5 μM Actinomycin D (ActD). In the middle, normalized distance of each locus from the nuclear periphery (the number of loci is indicated). All p-values calculated as in panel a but for ActD vs Control. (**c**) Same graphs as in panel a, but for hESC-CM obtained from the indicated gene edited hESC lines or wild-type (WT) control cells. Data from two independent cultures for all conditions but for RBM20 KO g2. All p-values calculated as in panel a but for each condition vs WT; * < 0.05. Source data are provided as a Source Data file.

Supplementary Figure 10

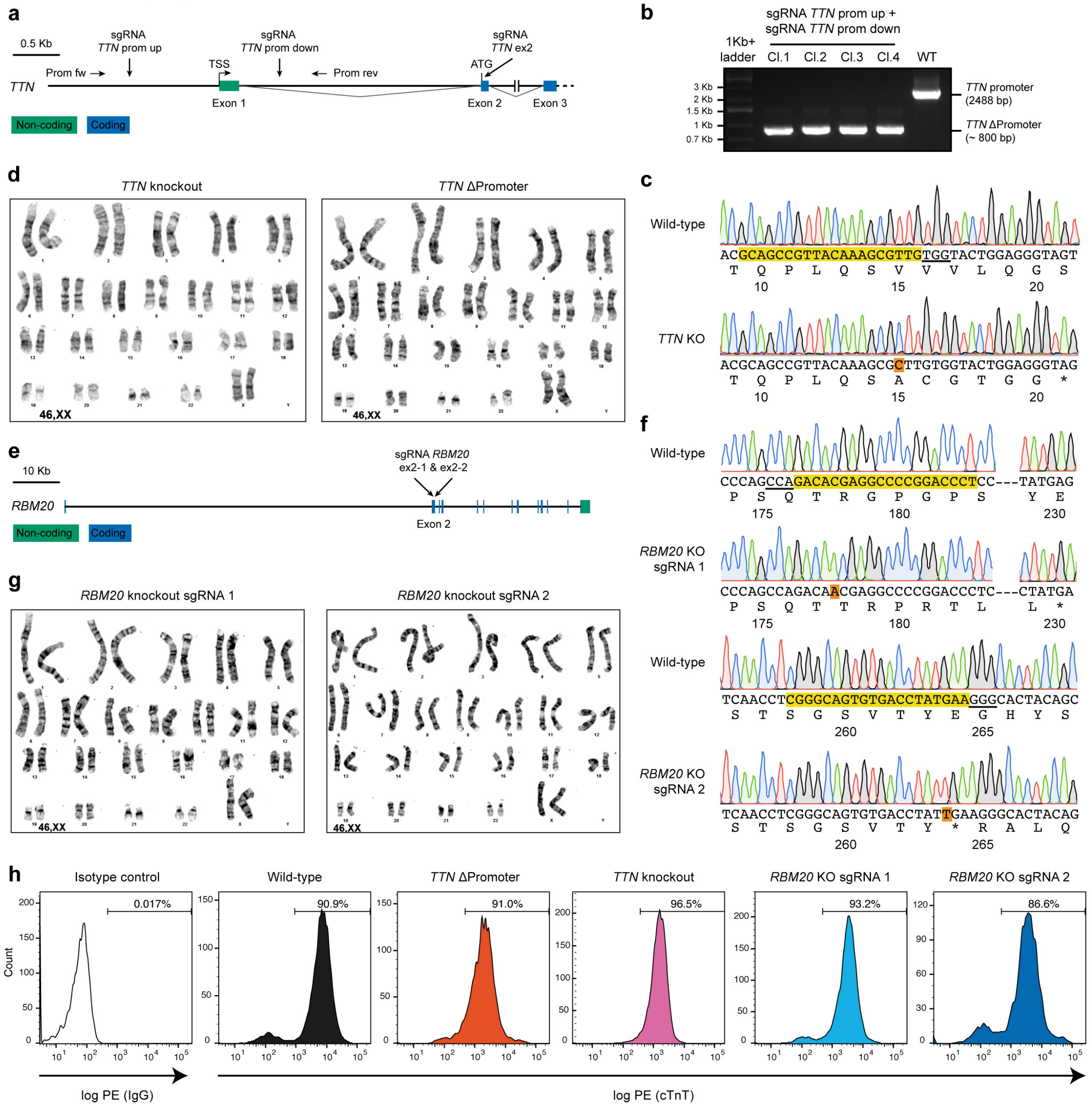

Supplementary Figure 10. Generation of gene edited hESC-CM to investigate the mechanisms regulating *TTN trans* interactions. (**a**) Schematic of the genomic region encompassing the *TTN* promoter and the first coding exons. The position of CRISPR/Cas9 single guide RNAs (sgRNAs) employed to delete the promoter or to induce a frameshift mutation in the first coding exon is indicated; arrows depict primers used for genomic PCR. (**b**) Genomic PCR for the *TTN* promoter in selected hESC clones (Cl.) isolated after CRISPR/Cas9-

mediated gene editing with the indicated sgRNA combination; wild type (WT) hESCs were used as positive control. The analysis confirms homozygous deletion of a ~1.7 Kb genomic region in all gene edited clones (*TTN* ∆Prom). (**c**) Electropherograms from Sanger sequencing of genomic PCR products from exon 2 of *TTN* in wild type or *TTN* knockout hESCs (KO; induced by sgRNA *TTN* ex2). The 20 bp-sgRNA targeting sequence is highlighted, and the protospacer adjacent motif (PAM) site is underlined A homozygous "C" insertion leads to a p.Val15AlafsTer21 mutation. (**d**) G-banding karyotype of *TTN* knockout and *TTN* ∆Prom hESCs; a normal 46 XX female karyotype is confirmed. (**e**) Schematic of the *RBM20* locus. The position of two sgRNAs employed to induce a frameshift mutation in the second exon is indicated. (**f**) As in c, but for genomic PCR products from exon 2 of *RBM20.* RBM20 knockout lines have homozygous insertions of a single nucleotide leading to p.Arg178ThrfsTer230 and p.Glu264Ter mutations for sgRNA1 and sgRNA2, respectively. (**g**) As in d, but for the two *RBM20* knockout hESC lines. (**h**) Representative flow cytometry for TNNT2 (cTnT) in cardiomyocytes derived from the indicated hESCs, indicating that all gene edited lines could be successfully differentiated in highly-pure cardiomyocytes.

Supplementary Figure 11

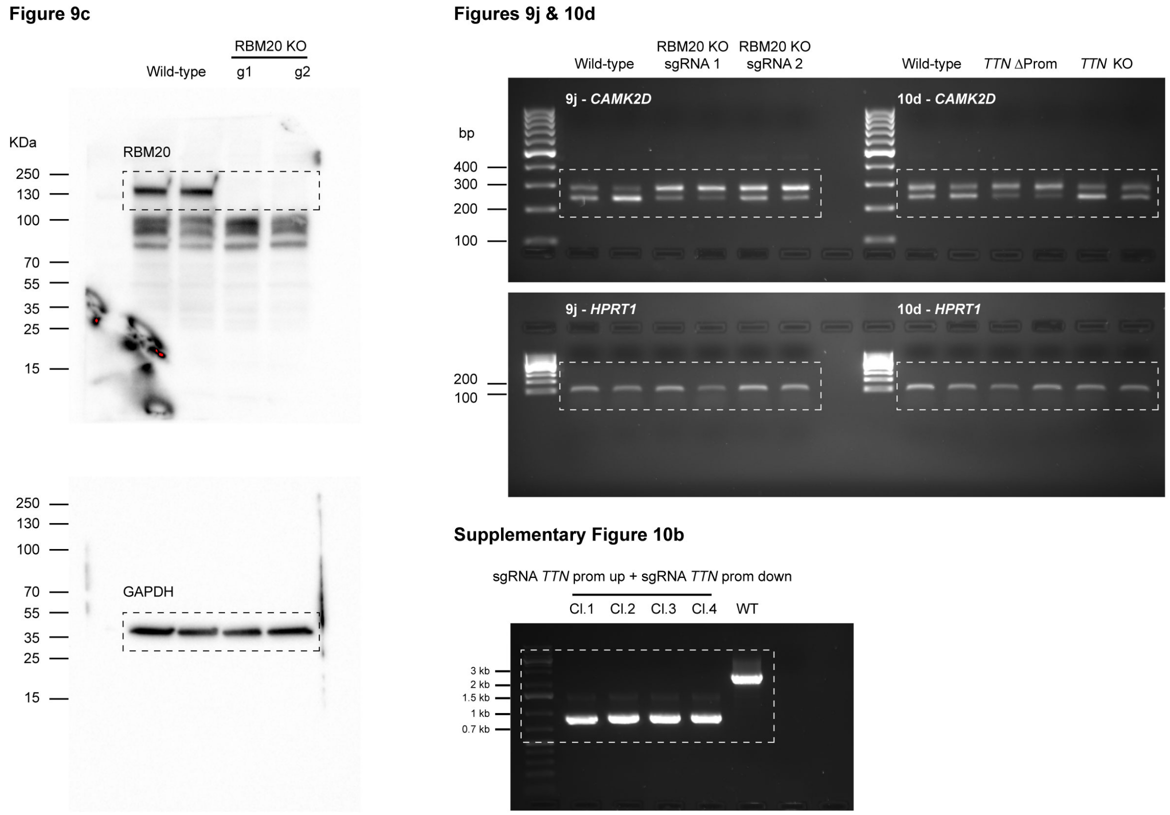

Supplementary Figure 11. Uncropped gel images. Unmodified Western blot and DNA agarose gel electrophoresis images from the indicated figure panels. The cropping masks are shown by dotted boxes, and molecular weights are indicated.

Supplementary Tables

Supplementary Table 1. Hi-C sequencing statistics.

	Raw Data				
Sample	Read Pairs	Mapped Reads 1	Percent Mapped R1	Mapped Reads 2	Percent Mapped R2
hESCs day 0 Rep 1	165646878	163319338	98.59%	160481970	96.88%
hESCs day 0 Rep 2	161233027	159088080	98.67%	156048718	96.78%
hESCs day 2 Rep 1	235241251	232370140	98.78%	227591982	96.75%
hESCs day 2 Rep 2	140440525	138771612	98.81%	136685754	97.33%
hESCs day 5 Rep 1	239177692	236107648	98.72%	231580364	96.82%
hESCs day 5 Rep 2	178553593	176841706	99.04%	173499069	97.17%
hESCs day 14 Rep 1	235786059	232336187	98.54%	228270169	96.81%
hESCs day 14 Rep 2	258136490	254785514	98.70%	250240626	96.94%
Fetal heart Rep 1	209940595	204117844	97.23%	194318323	92.56%
Fetal heart Rep 2	279397192	272799884	97.64%	256392944	91.77%
hiPSCs day 0 Rep 1	137583155	115208484	83.74%	126873906	92.22%
hiPSCs day 0 Rep 2	197884395	193934937	98.00%	186380999	94.19%
hiPSCs day 2 Rep 1	119401571	104593113	87.60%	110400095	92.46%
hiPSCs day 2 Rep 2	122590623	119611269	97.57%	115541207	94.25%
hiPSCs day 5 Rep 1	115824636	88588832	76.49%	104846287	90.52%
hiPSCs day 5 Rep 2	166481065	161744811	97.16%	156419674	93.96%
hiPSCs day 14 Rep 1	147673950	133751829	90.57%	135000005	91.42%
hiPSCs day 14 Rep 2	211902216	206829336	97.61%	200240212	94.50%

	After Filtering for Duplicates/Quality								
Sample	Valid Pairs	Unique Valid Pairs	Percent Unique	*Trans* (Total)	*Trans* %	*Cis* (Total)	*Cis* %	*Cis* (<20Kb)	*Cis* (>20Kb)
hESCs day 0 Rep 1	74574813	72979455	97.86%	22059973	30.23%	50919482	69.77%	12777607	38141875
hESCs day 0 Rep 2	68832201	65667042	95.40%	19810884	30.17%	45856158	69.83%	7510223	38345935
hESCs day 2 Rep 1	88968472	87365210	98.20%	44438155	50.86%	42927055	49.14%	9297132	33629923
hESCs day 2 Rep 2	57331090	56148676	97.94%	23459615	41.78%	32689061	58.22%	5961200	26727861
hESCs day 5 Rep 1	96971560	95048932	98.02%	49722758	52.31%	45326174	47.69%	11014371	34311803
hESCs day 5 Rep 2	69073141	66830739	96.75%	24569801	36.76%	42260938	63.24%	7358536	34902402
hESCs day 14 Rep 1	95818638	93788571	97.88%	53494591	57.04%	40293980	42.96%	12587671	27706309
hESCs day 14 Rep 2	94806156	91730323	96.76%	50950360	55.54%	40779963	44.46%	12039760	28740203
Fetal heart Rep 1	83524660	66895808	80.09%	29581537	44.22%	37314271	55.78%	4313170	33001101
Fetal heart Rep 2	110503532	86242067	78.04%	40146619	46.55%	46095448	53.45%	6514061	39581387
hiPSCs day 0 Rep 1	56988966	54007165	94.77%	16091054	29.79%	37916111	70.21%	7412822	30503289
hiPSCs day 0 Rep 2	82049882	76253186	92.94%	18834725	24.70%	57418461	75.30%	15490486	41927975
hiPSCs day 2 Rep 1	46946167	43441373	92.53%	12790263	29.44%	30651110	70.56%	7150684	23500426
hiPSCs day 2 Rep 2	50283768	47473565	94.41%	15527388	32.71%	31946177	67.29%	7738805	24207372
hiPSCs day 5 Rep 1	33667377	30960376	91.96%	12369964	39.95%	18590412	60.05%	5382365	13208047
hiPSCs day 5 Rep 2	64348377	60013698	93.26%	28398291	47.32%	31615407	52.68%	6284824	25330583
hiPSCs day 14 Rep 1	54948746	50984157	92.78%	26326245	51.64%	24657912	48.36%	6993099	17664813
hiPSCs day 14 Rep 2	96410011	90414067	93.78%	40724211	45.04%	49689856	54.96%	13430044	36259812

	Strand Read Distribution				QuASAR-QC 1Mb
Sample	FF	RR	RF	FR	
hESCs day 0 Rep 1	18635673	18635243	18558553	18745344	0.0488
hESCs day 0 Rep 2	17215042	17189902	17169868	17257389	0.0542
hESCs day 2 Rep 1	22235341	22236614	22163176	22333341	0.0442
hESCs day 2 Rep 2	14332789	14315174	14307817	14375310	0.0498
hESCs day 5 Rep 1	24230874	24225288	24163554	24351844	0.0463
hESCs day 5 Rep 2	17260014	17249463	17224706	17338958	0.0497
hESCs day 14 Rep 1	23932396	23944364	23811595	24130283	0.0400
hESCs day 14 Rep 2	23700423	23660467	23579174	23866092	0.0431
Fetal heart Rep 1	20885961	20878632	20876609	20883458	0.0484
Fetal heart Rep 2	27633225	27615787	27604297	27650223	0.0532
hiPSCs day 0 Rep 1	14242463	14244556	14207665	14294282	0.047365
hiPSCs day 0 Rep 2	20509684	20495012	20411689	20633497	0.04932
hiPSCs day 2 Rep 1	11733987	11721434	11686967	11803779	0.038117
hiPSCs day 2 Rep 2	12574627	12560001	12534576	12614564	0.039714
hiPSCs day 5 Rep 1	8417818	8400971	8362316	8486272	0.031701
hiPSCs day 5 Rep 2	16081633	16083106	16050471	16133167	0.040819
hiPSCs day 14 Rep 1	13734800	13734011	13660793	13819142	0.031748
hiPSCs day 14 Rep 2	24108482	24079493	24029660	24192376	0.043249

Supplementary Table 2. HiC-Rep scores between samples.

	ESC Rep1	ESC Rep2	MES Rep1	MES Rep2	CP Rep1	CP Rep2	CM Rep1	CM Rep2	FETAL Rep1	FETAL Rep2
**ESC Rep1**	1.000	0.897	0.939	0.885	0.906	0.846	0.760	0.605	0.802	0.767
**ESC Rep2**	0.897	1.000	0.845	0.729	0.736	0.672	0.566	0.429	0.753	0.754
**MES Rep1**	0.939	0.845	1.000	0.933	0.944	0.884	0.825	0.694	0.847	0.798
**MES Rep2**	0.885	0.729	0.933	1.000	0.955	0.957	0.886	0.794	0.736	0.639
**CP Rep1**	0.906	0.736	0.944	0.955	1.000	0.936	0.896	0.778	0.811	0.737
**CP Rep2**	0.846	0.672	0.884	0.957	0.936	1.000	0.890	0.806	0.704	0.602
**CM Rep1**	0.760	0.566	0.825	0.886	0.896	0.890	1.000	0.953	0.732	0.609
**CM Rep2**	0.605	0.429	0.694	0.794	0.778	0.806	0.953	1.000	0.595	0.448
**FETAL Rep1**	0.802	0.753	0.847	0.736	0.811	0.704	0.732	0.595	1.000	0.936
**FETAL Rep2**	0.767	0.754	0.798	0.639	0.737	0.602	0.609	0.448	0.936	1.000

Supplementary Table 3. RNA-seq sequencing statistics.

	Raw Data				
Sample	Read Pairs	Uniquely Mapped Pairs	Percent Uniquely Mapped	Pairs Mapped to Multiple Loci	Percent Mapped to Multiple Loci
Day 0 Rep 1	53692331	47109143	87.74%	2950649	5.50%
Day 0 Rep 2	51804420	46128530	89.04%	2572857	4.97%
Day 2 Rep 1	42767977	36828645	86.11%	2213374	5.18%
Day 2 Rep 2	49364876	44336824	89.81%	2466888	5.00%
Day 5 Rep 1	70802520	64030444	90.44%	3757341	5.31%
Day 5 Rep 2	58470047	52740164	90.20%	2884471	4.93%
Day 14 Rep 1	49032296	43191615	88.09%	3031221	6.18%
Day 14 Rep 2	64358359	58364375	90.69%	3419083	5.31%

Supplementary Table 4. Biological process gene ontology terms associated with differentially expressed genes peaking in cardiomyocyte stage.

Term	Count	PValue	Fold Enrichment	Benjamini
GO:0006091~generation of precursor metabolites and energy	170	2.41E-39	2.74	2.33E-35
GO:0015980~energy derivation by oxidation of organic compounds	137	9.64E-39	3.09	4.66E-35
GO:0045333~cellular respiration	103	4.85E-37	3.62	1.56E-33
GO:0022904~respiratory electron transport chain	74	2.64E-32	4.15	6.37E-29
GO:0055114~oxidation-reduction process	305	4.04E-32	1.90	7.81E-29
GO:0022900~electron transport chain	74	1.62E-31	4.08	2.60E-28
GO:0042775~mitochondrial ATP synthesis coupled electron transport	65	2.05E-30	4.37	2.83E-27
GO:0042773~ATP synthesis coupled electron transport	65	5.67E-30	4.32	6.84E-27
GO:0046034~ATP metabolic process	115	2.73E-29	2.90	2.93E-26
GO:0006119~oxidative phosphorylation	70	1.04E-28	3.97	1.00E-25
GO:0009205~purine ribonucleoside triphosphate metabolic process	121	1.27E-28	2.77	1.11E-25
GO:0009144~purine nucleoside triphosphate metabolic process	123	1.29E-28	2.75	1.04E-25
GO:0009199~ribonucleoside triphosphate metabolic process	121	1.52E-27	2.71	1.13E-24
GO:0009167~purine ribonucleoside monophosphate metabolic process	123	1.05E-26	2.64	7.21E-24
GO:0009126~purine nucleoside monophosphate metabolic process	123	1.53E-26	2.63	9.87E-24
GO:0044710~single-organism metabolic process	882	1.30E-25	1.33	7.86E-23
GO:0007507~heart development	179	1.99E-25	2.14	1.13E-22
GO:0009141~nucleoside triphosphate metabolic process	124	2.36E-25	2.55	1.27E-22
GO:0009161~ribonucleoside monophosphate metabolic process	123	8.79E-25	2.53	4.47E-22
GO:0006120~mitochondrial electron transport, NADH to ubiquinone	43	7.55E-24	4.96	3.65E-21
GO:0009123~nucleoside monophosphate metabolic process	125	8.42E-24	2.46	3.87E-21
GO:0046128~purine ribonucleoside metabolic process	136	2.18E-23	2.34	9.55E-21
GO:0042278~purine nucleoside metabolic process	136	5.36E-23	2.32	2.25E-20
GO:0009119~ribonucleoside metabolic process	139	2.94E-21	2.21	1.18E-18
GO:0010257~NADH dehydrogenase complex assembly	45	3.87E-21	4.37	1.50E-18
GO:0032981~mitochondrial respiratory chain complex I assembly	45	3.87E-21	4.37	1.50E-18
GO:0097031~mitochondrial respiratory chain complex I biogenesis	45	3.87E-21	4.37	1.50E-18
GO:0061061~muscle structure development	186	1.29E-20	1.93	4.80E-18
GO:0009116~nucleoside metabolic process	143	6.08E-20	2.12	2.17E-17

GO:0048738~cardiac muscle tissue development	83	2.32E-19	2.73	7.99E-17
GO:0033108~mitochondrial respiratory chain complex assembly	53	3.60E-19	3.60	1.20E-16
GO:1901657~glycosyl compound metabolic process	146	5.93E-19	2.05	1.91E-16
GO:0042692~muscle cell differentiation	128	8.63E-19	2.16	2.69E-16
GO:0003012~muscle system process	138	1.35E-18	2.09	4.07E-16
GO:0007005~mitochondrion organization	194	2.94E-18	1.82	8.60E-16
GO:0060537~muscle tissue development	126	2.35E-17	2.10	6.67E-15
GO:0044281~small molecule metabolic process	452	3.31E-17	1.42	9.14E-15
GO:0072359~circulatory system development	253	3.57E-17	1.64	9.58E-15
GO:0072358~cardiovascular system development	253	3.57E-17	1.64	9.58E-15
GO:0009062~fatty acid catabolic process	49	1.06E-16	3.45	2.90E-14
GO:0014706~striated muscle tissue development	120	2.69E-16	2.10	5.64E-14
GO:0051146~striated muscle cell differentiation	95	3.03E-16	2.33	8.25E-14
GO:0006941~striated muscle contraction	71	3.42E-16	2.70	8.04E-14
GO:0006793~phosphorus metabolic process	659	5.19E-16	1.31	1.31E-13
GO:0009150~purine ribonucleotide metabolic process	158	9.99E-16	1.86	2.30E-13
GO:0006163~purine nucleotide metabolic process	164	1.01E-15	1.83	2.24E-13
GO:0006796~phosphate-containing compound metabolic process	656	1.29E-15	1.30	2.92E-13
GO:0006996~organelle organization	774	2.63E-15	1.26	5.72E-13
GO:0006936~muscle contraction	113	2.77E-15	2.09	5.83E-13
GO:0055002~striated muscle cell development	62	2.82E-15	2.81	5.70E-13
GO:0006914~autophagy	133	4.47E-15	1.95	8.94E-13
GO:0072521~purine-containing compound metabolic process	168	5.86E-15	1.79	1.16E-12
GO:0030029~actin filament-based process	189	6.08E-15	1.72	1.18E-12
GO:0009117~nucleotide metabolic process	187	1.20E-14	1.72	2.27E-12
GO:0009259~ribonucleotide metabolic process	158	1.48E-14	1.81	2.74E-12
GO:0055001~muscle cell development	65	1.69E-14	2.65	3.08E-12
GO:0006753~nucleoside phosphate metabolic process	188	1.99E-14	1.70	3.55E-12
GO:0072329~monocarboxylic acid catabolic process	51	4.34E-14	3.00	7.62E-12
GO:0019693~ribose phosphate metabolic process	159	7.56E-14	1.77	1.30E-11
GO:0016310~phosphorylation	488	1.52E-13	1.34	2.58E-11
GO:0055086~nucleobase-containing small molecule metabolic process	196	1.85E-13	1.65	3.08E-11
GO:0016043~cellular component organization	1168	1.99E-13	1.17	3.26E-11
GO:0006635~fatty acid beta-oxidation	39	2.01E-13	3.46	3.24E-11
GO:0003007~heart morphogenesis	84	2.20E-13	2.23	3.48E-11
GO:0071495~cellular response to endogenous stimulus	288	2.82E-13	1.49	4.39E-11

GO:0055013~cardiac muscle cell development	35	3.17E-13	3.69	4.87E-11
GO:0019395~fatty acid oxidation	47	3.22E-13	3.03	4.87E-11
GO:0065009~regulation of molecular function	601	5.49E-13	1.28	8.15E-11
GO:0051641~cellular localization	542	6.15E-13	1.31	9.00E-11
GO:0006464~cellular protein modification process	770	7.54E-13	1.23	1.09E-10
GO:0036211~protein modification process	770	7.54E-13	1.23	1.09E-10
GO:0034440~lipid oxidation	47	8.36E-13	2.97	1.19E-10
GO:0044248~cellular catabolic process	369	9.23E-13	1.40	1.29E-10
GO:0003205~cardiac chamber development	63	1.41E-12	2.49	1.94E-10
GO:0009056~catabolic process	442	1.76E-12	1.34	2.40E-10
GO:1901575~organic substance catabolic process	419	1.77E-12	1.36	2.37E-10
GO:0055006~cardiac cell development	35	2.34E-12	3.51	3.10E-10
GO:0007034~vacuolar transport	96	2.49E-12	2.03	3.25E-10
GO:0043436~oxoacid metabolic process	213	2.86E-12	1.57	3.69E-10
GO:0044267~cellular protein metabolic process	953	3.70E-12	1.19	4.70E-10
GO:0009719~response to endogenous stimulus	353	3.83E-12	1.40	4.80E-10
GO:0009060~aerobic respiration	35	4.38E-12	3.45	5.42E-10
GO:0019752~carboxylic acid metabolic process	211	5.18E-12	1.56	6.33E-10
GO:0019637~organophosphate metabolic process	267	6.27E-12	1.47	7.58E-10
GO:0060048~cardiac muscle contraction	52	9.16E-12	2.65	1.09E-09
GO:0008104~protein localization	518	1.02E-11	1.29	1.20E-09
GO:0006082~organic acid metabolic process	226	1.16E-11	1.52	1.34E-09
GO:0071840~cellular component organization or biogenesis	1180	1.51E-11	1.15	1.74E-09
GO:0044242~cellular lipid catabolic process	68	1.60E-11	2.29	1.82E-09
GO:0090257~regulation of muscle system process	72	1.67E-11	2.23	1.87E-09
GO:0003206~cardiac chamber morphogenesis	51	1.67E-11	2.64	1.85E-09
GO:0055007~cardiac muscle cell differentiation	45	1.73E-11	2.84	1.90E-09
GO:0016236~macroautophagy	73	2.17E-11	2.20	2.36E-09
GO:0030239~myofibril assembly	31	2.70E-11	3.58	2.90E-09
GO:0016197~endosomal transport	84	3.39E-11	2.06	3.60E-09
GO:0046907~intracellular transport	356	5.04E-11	1.37	5.29E-09
GO:0030163~protein catabolic process	204	5.37E-11	1.54	5.58E-09
GO:0032787~monocarboxylic acid metabolic process	152	5.63E-11	1.66	5.79E-09
GO:0070252~actin-mediated cell contraction	45	6.04E-11	2.75	6.14E-09
GO:0006631~fatty acid metabolic process	110	6.46E-11	1.84	6.50E-09

Supplementary Table 5. Biological process gene ontology terms associated with differentially expressed genes peaking in cardiomyocytes in regions of the genome that transition B to A.

Term	Count	PValue	Fold Enrichment	Benjamini
GO:0072359~circulatory system development	39	2.06E-10	3.24	8.76E-07
GO:0072358~cardiovascular system development	39	2.06E-10	3.24	8.76E-07
GO:0007507~heart development	28	3.57E-10	4.30	7.60E-07
GO:0003012~muscle system process	24	2.02E-09	4.65	2.87E-06
GO:0060537~muscle tissue development	22	9.30E-09	4.70	9.89E-06
GO:0048738~cardiac muscle tissue development	15	1.11E-07	6.33	9.41E-05
GO:0006928~movement of cell or subcellular component	50	1.54E-07	2.19	1.09E-04
GO:0001568~blood vessel development	25	3.26E-07	3.41	1.98E-04
GO:0035051~cardiocyte differentiation	12	4.33E-07	7.72	2.30E-04
GO:0044057~regulation of system process	23	4.74E-07	3.58	2.24E-04
GO:0009888~tissue development	48	5.26E-07	2.14	2.24E-04
GO:0014706~striated muscle tissue development	19	5.41E-07	4.26	2.09E-04
GO:0007399~nervous system development	55	6.11E-07	1.99	2.17E-04
GO:0051239~regulation of multicellular organismal process	63	6.56E-07	1.86	2.15E-04
GO:0048731~system development	88	6.59E-07	1.61	2.00E-04
GO:0044707~single-multicellular organism process	111	7.53E-07	1.47	2.14E-04
GO:0001944~vasculature development	25	9.09E-07	3.22	2.42E-04
GO:0006936~muscle contraction	18	1.15E-06	4.27	2.89E-04
GO:0090257~regulation of muscle system process	14	1.50E-06	5.55	3.55E-04
GO:0061061~muscle structure development	24	1.77E-06	3.20	3.97E-04
GO:0008015~blood circulation	22	1.99E-06	3.41	4.23E-04
GO:0003013~circulatory system process	22	2.24E-06	3.38	4.54E-04
GO:0060419~heart growth	9	3.50E-06	9.81	6.78E-04
GO:0030154~cell differentiation	75	8.17E-06	1.61	1.51E-03
GO:0055013~cardiac muscle cell development	8	8.32E-06	10.82	1.47E-03
GO:0044763~single-organism cellular process	183	9.23E-06	1.17	1.57E-03
GO:1901698~response to nitrogen compound	29	9.33E-06	2.54	1.53E-03
GO:0003300~cardiac muscle hypertrophy	8	1.05E-05	10.46	1.65E-03
GO:0048870~cell motility	37	1.15E-05	2.18	1.74E-03
GO:0051674~localization of cell	37	1.15E-05	2.18	1.74E-03
GO:0055006~cardiac cell development	8	1.17E-05	10.29	1.71E-03
GO:0048468~cell development	48	1.22E-05	1.91	1.73E-03
GO:0007275~multicellular organism development	92	1.29E-05	1.48	1.77E-03
GO:0014897~striated muscle hypertrophy	8	1.30E-05	10.12	1.73E-03

GO:0042692~muscle cell differentiation	17	1.60E-05	3.68	2.07E-03
GO:0014896~muscle hypertrophy	8	1.98E-05	9.51	2.47E-03
GO:0009653~anatomical structure morphogenesis	58	2.14E-05	1.73	2.59E-03
GO:0055017~cardiac muscle tissue growth	8	2.18E-05	9.37	2.58E-03
GO:0048646~anatomical structure formation involved in morphogenesis	33	2.24E-05	2.25	2.57E-03
GO:2000026~regulation of multicellular organismal development	43	2.39E-05	1.96	2.67E-03
GO:1901699~cellular response to nitrogen compound	21	2.98E-05	2.95	3.25E-03
GO:0048869~cellular developmental process	78	3.04E-05	1.53	3.23E-03
GO:0061337~cardiac conduction	9	3.23E-05	7.28	3.35E-03
GO:0071495~cellular response to endogenous stimulus	33	3.95E-05	2.19	3.99E-03
GO:0048699~generation of neurons	36	4.24E-05	2.08	4.19E-03
GO:0008016~regulation of heart contraction	13	4.39E-05	4.38	4.23E-03
GO:0030029~actin filament-based process	23	4.65E-05	2.69	4.39E-03
GO:0048856~anatomical structure development	98	5.04E-05	1.41	4.66E-03
GO:0044767~single-organism developmental process	98	5.12E-05	1.40	4.63E-03
GO:0009719~response to endogenous stimulus	39	5.55E-05	1.98	4.91E-03
GO:0055002~striated muscle cell development	10	5.64E-05	5.81	4.89E-03
GO:0042391~regulation of membrane potential	16	5.82E-05	3.49	4.94E-03
GO:0048513~animal organ development	64	6.62E-05	1.60	5.51E-03
GO:0010243~response to organonitrogen compound	25	6.63E-05	2.49	5.42E-03
GO:0022008~neurogenesis	37	6.85E-05	2.01	5.49E-03
GO:0051270~regulation of cellular component movement	25	8.54E-05	2.45	6.71E-03
GO:0051146~striated muscle cell differentiation	13	8.64E-05	4.08	6.66E-03
GO:0050793~regulation of developmental process	48	8.96E-05	1.77	6.79E-03
GO:0016477~cell migration	32	9.36E-05	2.12	6.96E-03
GO:0040011~locomotion	38	9.64E-05	1.95	7.05E-03
GO:0032502~developmental process	99	9.76E-05	1.38	7.02E-03
GO:0045595~regulation of cell differentiation	38	1.16E-04	1.93	8.21E-03
GO:0055001~muscle cell development	10	1.27E-04	5.23	8.84E-03
GO:0032501~multicellular organismal process	117	1.28E-04	1.31	8.72E-03
GO:0003231~cardiac ventricle development	9	1.30E-04	5.98	8.76E-03
GO:0060048~cardiac muscle contraction	9	1.46E-04	5.89	9.69E-03
GO:0060038~cardiac muscle cell proliferation	6	1.62E-04	11.48	1.05E-02
GO:0003205~cardiac chamber development	10	1.63E-04	5.06	1.05E-02
GO:0033002~muscle cell proliferation	10	1.63E-04	5.06	1.05E-02
GO:0086001~cardiac muscle cell action potential	7	1.72E-04	8.45	1.09E-02
GO:0003007~heart morphogenesis	12	1.76E-04	4.09	1.09E-02

GO:0048514~blood vessel morphogenesis	18	1.81E-04	2.88	1.11E-02
GO:0014742~positive regulation of muscle hypertrophy	5	1.84E-04	17.06	1.12E-02
GO:0010613~positive regulation of cardiac muscle hypertrophy	5	1.84E-04	17.06	1.12E-02
GO:1901700~response to oxygen-containing compound	36	1.96E-04	1.93	1.17E-02
GO:0003008~system process	45	2.01E-04	1.75	1.18E-02
GO:0060284~regulation of cell development	25	2.10E-04	2.31	1.21E-02
GO:0006941~striated muscle contraction	10	2.17E-04	4.87	1.24E-02
GO:0090130~tissue migration	12	2.28E-04	3.97	1.28E-02
GO:0001667~ameboidal-type cell migration	14	2.33E-04	3.43	1.30E-02
GO:0055007~cardiac muscle cell differentiation	8	2.34E-04	6.47	1.29E-02
GO:0090075~relaxation of muscle	5	2.58E-04	15.69	1.40E-02
GO:0003208~cardiac ventricle morphogenesis	7	3.02E-04	7.63	1.62E-02
GO:0044699~single-organism process	190	3.08E-04	1.11	1.63E-02
GO:0086065~cell communication involved in cardiac conduction	6	3.12E-04	10.02	1.62E-02
GO:0010644~cell communication by electrical coupling	5	3.51E-04	14.53	1.81E-02
GO:0035265~organ growth	9	3.81E-04	5.12	1.93E-02
GO:0071417~cellular response to organonitrogen compound	17	3.97E-04	2.80	1.99E-02
GO:0061138~morphogenesis of a branching epithelium	10	4.37E-04	4.43	2.16E-02
GO:1903522~regulation of blood circulation	13	4.64E-04	3.40	2.27E-02
GO:0014855~striated muscle cell proliferation	6	5.02E-04	9.05	2.42E-02
GO:0007417~central nervous system development	25	5.04E-04	2.17	2.41E-02
GO:1901701~cellular response to oxygen-containing compound	25	5.71E-04	2.15	2.70E-02
GO:0044091~membrane biogenesis	5	6.05E-04	12.66	2.82E-02
GO:0060562~epithelial tube morphogenesis	13	6.06E-04	3.30	2.80E-02
GO:0035637~multicellular organismal signaling	9	6.07E-04	4.77	2.77E-02
GO:0044700~single organism signaling	103	6.68E-04	1.30	3.01E-02
GO:0010631~epithelial cell migration	11	6.80E-04	3.79	3.03E-02
GO:0071869~response to catecholamine	5	6.85E-04	12.26	3.02E-02
GO:0071867~response to monoamine	5	6.85E-04	12.26	3.02E-02

Supplementary Table 6. Jaccard index scores for TAD boundary overlap.

DI Method Jaccard								
	ESC Rep1	ESC Rep2	MES Rep1	MES Rep2	CP Rep1	CP Rep2	CM Rep1	CM Rep2
ESC Rep1	1	0.6179577	0.6138462	0.58373	0.6004372	0.6012752	0.5621026	0.5135478
ESC Rep2	0.6179577	1	0.6131491	0.5999526	0.5763937	0.5428192	0.5412324	0.5137065
MES Rep1	0.6138462	0.6131491	1	0.6487543	0.6323777	0.6079742	0.6043046	0.5397048
MES Rep2	0.58373	0.5999526	0.6487543	1	0.6300774	0.5935706	0.5765116	0.5238317
CP Rep1	0.6004372	0.5763937	0.6323777	0.6300774	1	0.6351034	0.6087678	0.5532971
CP Rep2	0.6012752	0.5428192	0.6079742	0.5935706	0.6351034	1	0.5900114	0.5418372
CM Rep1	0.56210259	0.54123244	0.60430464	0.57651163	0.60876777	0.59001135	1	0.62993097
CM Rep2	0.51354784	0.51370652	0.53970485	0.52383167	0.55329709	0.5418372	0.62993097	1

Insulation Score Jaccard								
	ESC Rep1	ESC Rep2	MES Rep1	MES Rep2	CP Rep1	CP Rep2	CM Rep1	CM Rep2
ESC Rep1	1	0.832497	0.8104142	0.7843458	0.7760429	0.7679777	0.7506868	0.7337574
ESC Rep2	0.832497	1	0.7912482	0.7706509	0.752988	0.7430312	0.7187715	0.7065814
MES Rep1	0.8104142	0.7912482	1	0.8397736	0.8110599	0.7939116	0.763375	0.7421839
MES Rep2	0.7843458	0.7706509	0.8397736	1	0.8056297	0.7679083	0.7595474	0.7347604
CP Rep1	0.7760429	0.752988	0.8110599	0.8056297	1	0.8441785	0.7750774	0.7624467
CP Rep2	0.7679777	0.7430312	0.7939116	0.7679083	0.8441785	1	0.790198	0.7679083
CM Rep1	0.75068681	0.71877149	0.76337497	0.75954738	0.77507744	0.79019797	1	0.85611153
CM Rep2	0.73375738	0.70658135	0.74218385	0.73476035	0.76244666	0.76790831	0.85611153	1

Supplementary Table 7. ATAC-seq sequencing statistics.

	Raw Data					After Filtering for Duplicates/Quality			
Sample	Read Pairs	Mapped	% Mapped	Mitochondria Reads	% Mitochondria	Non-Mitochondria	Mapped Reads	Mitochondria Reads	% Mitochondria
Day 0 Rep 1	98288432	96358506	98.04%	38702151	40.16%	57656355	47633411	1998436	4.20%
Day 0 Rep 2	192101258	188002217	97.87%	93355966	49.66%	94646251	73531448	2462055	3.35%
Day 2 Rep 1	97441630	95005024	97.50%	31310401	32.96%	63694623	52785208	1730677	3.28%
Day 2 Rep 2	140863604	137402984	97.54%	59047777	42.97%	78355207	63065226	2106549	3.34%
Day 5 Rep 1	92290768	89459783	96.93%	23765951	26.57%	65693832	55241696	1524349	2.76%
Day 5 Rep 2	117570914	115551442	98.28%	51659427	44.71%	63892015	52235443	2735768	5.24%
Day 14 Rep 1	148082788	144502413	97.58%	58219720	40.29%	86282693	69667225	2425594	3.48%
Day 14 Rep 2	83006516	81391248	98.05%	35320785	43.40%	46070463	38343921	2186209	5.70%

	Peak Quality Metrics		
Sample	Peaks Detected	Reads in Peak	FRIP
Day 0 Rep 1	103189	9448934	0.2071
Day 0 Rep 2	156234	19467098	0.2739
Day 2 Rep 1	108425	12539240	0.2456
Day 2 Rep 2	156282	18640214	0.3058
Day 5 Rep 1	99889	10044237	0.1870
Day 5 Rep 2	120247	13383746	0.2704
Day 14 Rep 1	92891	10835040	0.1611
Day 14 Rep 2	55873	4937025	0.1365

Supplementary Table 8. Biological process gene ontology terms associated with differentially expressed genes peaking in cardiomyocyte stage and *trans*-associated with *TTN*.

Term	Count	PValue	Fold Enrichment	Benjamini
GO:0007034~vacuolar transport	13	7.67E-04	3.21	9.46E-01
GO:0061024~membrane organization	28	1.13E-03	1.95	8.83E-01
GO:0006941~striated muscle contraction	9	1.91E-03	3.99	9.12E-01
GO:0086001~cardiac muscle cell action potential	6	2.11E-03	6.60	8.66E-01
GO:0007166~cell surface receptor signaling pathway	55	2.47E-03	1.47	8.49E-01
GO:0016197~endosomal transport	11	2.73E-03	3.14	8.24E-01
GO:0006900~membrane budding	7	3.68E-03	4.72	8.66E-01
GO:0007267~cell-cell signaling	35	3.82E-03	1.65	8.38E-01
GO:0010038~response to metal ion	12	4.06E-03	2.78	8.21E-01
GO:0016043~cellular component organization	106	4.39E-03	1.24	8.13E-01
GO:0015980~energy derivation by oxidation of organic compounds	11	4.82E-03	2.90	8.12E-01
GO:0009144~purine nucleoside triphosphate metabolic process	11	5.19E-03	2.87	8.08E-01
GO:0055114~oxidation-reduction process	25	5.41E-03	1.82	7.96E-01
GO:0044763~single-organism cellular process	189	5.47E-03	1.10	7.75E-01
GO:0007399~nervous system development	45	6.40E-03	1.48	8.04E-01
GO:0060048~cardiac muscle contraction	7	6.71E-03	4.17	7.99E-01
GO:0006091~generation of precursor metabolites and energy	13	6.96E-03	2.45	7.91E-01
GO:0071840~cellular component organization or biogenesis	107	7.08E-03	1.22	7.78E-01
GO:0086003~cardiac muscle cell contraction	5	7.19E-03	6.50	7.65E-01
GO:0016050~vesicle organization	11	8.57E-03	2.66	8.06E-01
GO:0065008~regulation of biological quality	66	9.02E-03	1.33	8.07E-01
GO:0009141~nucleoside triphosphate metabolic process	11	9.03E-03	2.65	7.92E-01
GO:0048514~blood vessel morphogenesis	15	9.13E-03	2.18	7.81E-01
GO:0044087~regulation of cellular component biogenesis	21	9.25E-03	1.86	7.71E-01
GO:0002027~regulation of heart rate	6	9.28E-03	4.66	7.59E-01
GO:0007169~transmembrane receptor protein tyrosine kinase signaling pathway	18	9.48E-03	1.98	7.52E-01
GO:0006996~organelle organization	69	9.54E-03	1.31	7.42E-01
GO:0016192~vesicle-mediated transport	33	9.57E-03	1.58	7.30E-01
GO:0030048~actin filament-based movement	7	1.05E-02	3.79	7.50E-01
GO:0000045~autophagosome assembly	5	1.09E-02	5.76	7.52E-01
GO:0022607~cellular component assembly	51	1.12E-02	1.39	7.50E-01
GO:0061337~cardiac conduction	6	1.15E-02	4.42	7.48E-01

GO:0030838~positive regulation of actin filament polymerization	6	1.15E-02	4.42	7.48E-01
GO:0030258~lipid modification	10	1.15E-02	2.73	7.38E-01
GO:0060411~cardiac septum morphogenesis	5	1.15E-02	5.67	7.27E-01
GO:0003012~muscle system process	13	1.16E-02	2.29	7.19E-01
GO:1901215~negative regulation of neuron death	8	1.18E-02	3.25	7.16E-01
GO:1905037~autophagosome organization	5	1.22E-02	5.58	7.16E-01
GO:0086091~regulation of heart rate by cardiac conduction	4	1.26E-02	8.17	7.19E-01
GO:0009205~purine ribonucleoside triphosphate metabolic process	10	1.28E-02	2.68	7.15E-01
GO:0016054~organic acid catabolic process	9	1.28E-02	2.90	7.07E-01
GO:0072593~reactive oxygen species metabolic process	9	1.28E-02	2.90	7.07E-01
GO:0070252~actin-mediated cell contraction	6	1.30E-02	4.29	7.04E-01
GO:0040011~locomotion	33	1.32E-02	1.54	7.02E-01
GO:0001525~angiogenesis	13	1.32E-02	2.25	6.93E-01
GO:0007167~enzyme linked receptor protein signaling pathway	23	1.42E-02	1.72	7.10E-01
GO:0016055~Wnt signaling pathway	14	1.44E-02	2.14	7.07E-01
GO:0034613~cellular protein localization	34	1.44E-02	1.52	7.00E-01
GO:0009199~ribonucleoside triphosphate metabolic process	10	1.45E-02	2.62	6.94E-01
GO:0198738~cell-cell signaling by wnt	14	1.46E-02	2.13	6.90E-01

Supplementary Table 9. Primer sequences.

Target	Forward primer (5'-3')	Reverse primer (5'-3')	Amplicon (bp)	Application
CACNA1C. (all isoforms)	GCCGCTGCAGGAGAGTTTTA	CCCACATGTGCAAGACCACA	151	RT-qPCR
CACNA1C ex9*-10	CGGGCATGCTTGATCAGAAG	CCGCAGTTTTCTCCCTCGAT	142	RT-qPCR
CAMK2D ex12-19	CAACTATGCTGGCTACAAGGAA	CCCAAAGCTTCAGGTTCAAA	Variable (241-343)	RT-PCR
GATA4	ACACCCCAATCTCGATATGTTTG	GTTGCACAGATAGTGACCCGT	115	RT-qPCR
HPRT1	TGACACTGGCAAAACAATGCA	GGTCCTTTTCACCAGCAAGCT	94	RT-qPCR; RT-PCR
KDR	GTGATCGGAAATGACACTGGAG	CATGTTGGTCACTAACAGAAGCA	124	RT-qPCR
MYH6	GCCCTTTGACATTCGCACTG	GGTTTCAGCAATGACCTTGCC	103	RT-qPCR
NANOG	TTTGTGGGCCTGAAGAAAACT	AGGGCTGTCCTGAATAAGCAG	116	RT-qPCR
PDGFRA	CCGGCGTTCCTGGTCTTAG	GCTCACTTCACTCTCCCCAAAG	153	RT-qPCR
POU5F1	GGGTTCTATTTGGGAAGGTAT	TTCATTGTTGTCAGCTTCCT	131	RT-qPCR
RBM20	AAGGCCAAGCAGAATGAGAA	CCCTCCTGTTCCTCTTTTCC	119	RT-qPCR
RPLP0	GGCGTCCTCGTGGAAGTGAC	GCCTTGCGCATCATGGTGTT	254	RT-qPCR
T	CAAATCCTCATCCTCAGTTTG	GTCAGAATAGGTTGGAGAATTG	143	RT-qPCR
TNNT2	TTCACCAAAGATCTGCTCCTCGCT	TTATTACTGGTGTGGAGTGGGTGTGG	166	RT-qPCR
TTN (all isoforms)	GTAAAAAGAGCTGCCCCAGTGA	GCTAGGTGGCCCAGTGCTACT	68	RT-qPCR
TTN N2B	CCATGAGTATGGCAGTGTCA	TACGTTCCGGAAGTAATTTGC	92	RT-qPCR
TTN N2BA	GCCACAGTAACTGTGACAGAGG	GGCTGCCTTACCCACAAAAG	84	RT-qPCR
TTN N2BA-G	CCACAGCCTGAAGAAATACCA	TACGTTCCGGAAGTAATTTGC	253	RT-qPCR

Supplementary References

1. Kreitzer, F. R. *et al.* A robust method to derive functional neural crest cells from human pluripotent stem cells. *Am. J. Stem Cells* **2,** 119–31 (2013).
2. Ortmann, D. & Vallier, L. Variability of human pluripotent stem cell lines. *Curr. Opin. Genet. Dev.* **46,** 179–185 (2017).
