# Supplementary material for: Dynamics of genome reorganization during human cardiogenesis reveal an RBM20-dependent splicing factory
Source: Nat Commun. 2019 Apr 4;10:1538. doi: 10.1038/s41467-019-09483-5 (PMC6449405; doi:10.1038/s41467-019-09483-5)
Supplement: Supplementary file 3 — Reporting Summary [file 41467_2019_9483_MOESM3_ESM.pdf]

nature research

Corresponding author(s): Charles E .Murry

Reporting Summary

Nature Research wishes to improve the reproducibility of the work that we publish. This form provides structure for consistency and transparency in reporting. For further information on Nature Research policies, see Authors & Referees and the Editorial Policy Checklist.

Statistical parameters

When statistical analyses are reported, confirm that the following items are present in the relevant location (e.g. figure legend, table legend, main text, or Methods section).

n/a	Confirmed	
☐	☒	The exact sample size (n) for each experimental group/condition, given as a discrete number and unit of measurement
☐	☒	An indication of whether measurements were taken from distinct samples or whether the same sample was measured repeatedly
☐	☒	The statistical test(s) used AND whether they are one- or two-sided<br>*Only common tests should be described solely by name; describe more complex techniques in the Methods section.*
☒	☐	A description of all covariates tested
☐	☒	A description of any assumptions or corrections, such as tests of normality and adjustment for multiple comparisons
☐	☒	A full description of the statistics including central tendency (e.g. means) or other basic estimates (e.g. regression coefficient) AND variation (e.g. standard deviation) or associated estimates of uncertainty (e.g. confidence intervals)
☐	☒	For null hypothesis testing, the test statistic (e.g. F, t, r) with confidence intervals, effect sizes, degrees of freedom and P value noted<br>*Give P values as exact values whenever suitable.*
☒	☐	For Bayesian analysis, information on the choice of priors and Markov chain Monte Carlo settings
☒	☐	For hierarchical and complex designs, identification of the appropriate level for tests and full reporting of outcomes
☒	☐	Estimates of effect sizes (e.g. Cohen's d, Pearson's r), indicating how they were calculated
☐	☒	Clearly defined error bars<br>*State explicitly what error bars represent (e.g. SD, SE, CI)*

Our web collection on statistics for biologists may be useful.

Software and code

Policy information about availability of computer code

Data collection	No custom software was used for data collection.
Data analysis	All of the software used for the analyses presented is described in detail in the relevant Methods section, which also describes the relevant parameters used when these were not the default. The software used was: STAR, Cufflinks, BWA-MEM, Cytoscape, IGV, David, R/Bioconductor, DESeq2, DREME, Imaris, FACSDIVA, FlowJo. Custom scripts are available on github at https://github.com/pfields8/Fields_et_al_2018/.

For manuscripts utilizing custom algorithms or software that are central to the research but not yet described in published literature, software must be made available to editors/reviewers upon request. We strongly encourage code deposition in a community repository (e.g. GitHub). See the Nature Research guidelines for submitting code & software for further information.

Data

Policy information about availability of data

All manuscripts must include a data availability statement. This statement should provide the following information, where applicable:

- Accession codes, unique identifiers, or web links for publicly available datasets
- A list of figures that have associated raw data
- A description of any restrictions on data availability

All data for Hi-C, RNA-seq and ATAC-seq is available on Gene Expression Omnibus accession number GSE106690.

Field-specific reporting

Please select the best fit for your research. If you are not sure, read the appropriate sections before making your selection.

☒ Life sciences ☐ Behavioural & social sciences ☐ Ecological, evolutionary & environmental sciences

For a reference copy of the document with all sections, see nature.com/authors/policies/ReportingSummary-flat.pdf

Life sciences study design

All studies must disclose on these points even when the disclosure is negative.

Sample size	No power calculations were performed. Sample size was determined depending on the experiment type based on what is standard practice in the field of pluripotent stem cell biology to statically examine a large effect within an in vitro system which experiences only limited biological variability (see Figure Legends).
Data exclusions	No data points were excluded from any of the analyses presented.
Replication	All of the presented experiments were successfully reproduced, and the number of replications is indicated in the Methods and Figure Legends.
Randomization	No randomization was performed since this was not relevant to the study.
Blinding	No blinding was performed. This was deemed unnecessary since none of the analyses reported involved procedures that could be influenced by investigator bias (such as manual counting/measuring and/or morphological assessments). Indeed, all analyses presented involved automated processing of data through experimental instrumentation and/or automated computing procedures (including FISH experiments; see the Methods)

Reporting for specific materials, systems and methods

Materials & experimental systems

n/a	Involved in the study
☒	☐ Unique biological materials
☐	☒ Antibodies
☐	☒ Eukaryotic cell lines
☒	☐ Palaeontology
☒	☐ Animals and other organisms
☒	☐ Human research participants

Methods

n/a	Involved in the study
☒	☐ ChIP-seq
☐	☒ Flow cytometry
☒	☐ MRI-based neuroimaging

Antibodies

Antibodies used	Anti-cTnT mouse monoclonal [clone 13-11] (ThermoFisher MS-295); mouse non-immune IgG1 (eBioscience 14-4714); anti-RBM20 rabbit polyclonal (ThermoFisher PA5-57404); anit-GAPDH mouse monoclonal [clone 6C5] (Abcam 8245); anti-sarcomerinc α-actinin mouse monoclonal [clone EA-53] (Abcam ab9465); anti-Titin rabbit polyclonal (Myomedix; #Z1Z2)
Validation	All antibodies used have been validated by the suppliers for the applications for which they were used, and have been cited in multiple publications.

Eukaryotic cell lines

Policy information about cell lines

Cell line source(s)	The RUES2 hESC line was obtained from Rockefeller University and is available from WiCell. The WTC-11 hiPSC line was obtained from Coriell (GM25256;)
Authentication	The cell lines were tested to be karyotypically normal.
Mycoplasma contamination	Cells were tested by IDEXX and came back negative for known pathogens including mycoplasma.
Commonly misidentified lines (See ICLAC register)	No commonly misidentified cell lines were used.

Flow Cytometry

Plots

Confirm that:

- [x] The axis labels state the marker and fluorochrome used (e.g. CD4-FITC).
- [x] The axis scales are clearly visible. Include numbers along axes only for bottom left plot of group (a 'group' is an analysis of identical markers).
- [ ] All plots are contour plots with outliers or pseudocolor plots.
- [x] A numerical value for number of cells or percentage (with statistics) is provided.

Methodology

Sample preparation	hESC-derived CMs were collected and fixed in 4% Para-formaldehyde for 10 min. Cells were permeabilized with 0.75% Saponin in 5% FBS/PBS and stained with primary antibody for cTNT or isotype IgG1 control. Secondary antibody staining was anti-mouse PE. Cells were then stored in 5% FBS/PBS for analysis.
Instrument	Analysis was done on a BD FACSCanto II
Software	Data Collection: BD FACSDIVA. Data Analysis: FlowJo version 10.
Cell population abundance	No cell sorting was done in this study.
Gating strategy	Cells were first gated on the basis of forward and side scatter properties for single cells. An isotype negative control was used to determine the non-specific fluorescence, and positive cells were quantified by setting a boundary so that less than 1% of the isotype-only control cells would be considered positive.

- [x] Tick this box to confirm that a figure exemplifying the gating strategy is provided in the Supplementary Information.
